# Patterns of tobacco consumption among residents of a rural settlement: a cross-sectional study

**DOI:** 10.11606/S1518-8787.2017051006781

**Published:** 2017-11-13

**Authors:** Andrécia Cósmem da Silva, Lorena Silva Vargas, Roselma Lucchese, Bruno de Souza Calixto, Rafael Alves Guimarães, Ivania Vera, Paulo Alexandre de Castro, Valéria Pagotto, Inaina Lara Fernandes

**Affiliations:** I Universidade Estadual de Goiás. Ipameri, GO, Brasil; II Secretaria Municipal de Saúde de Catalão. Programa Municipal de Controle do Tabagismo. Catalão, GO, Brasil; III Universidade Federal de Goiás. Departamento de Enfermagem. Catalão, GO, Brasil; IV Hospital Nars Faiad. Catalão, GO, Brasil; V Universidade Federal de Goiás. Goiânia, GO, Brasil; VI Universidade Federal de Goiás. Departamento de Enfermagem. Catalão, GO, Brasil; VII Universidade Federal de Goiás. Departamento de Física. Catalão, GO, Brasil; VIII Secretaria Municipal de Saúde de Catalão. Catalão, GO, Brasil

**Keywords:** Tobacco Use Disorder, epidemiology, Rural Settlements, Risk Factors, Socioeconomic Factors, Cross-Sectional Studies, Tabagismo, epidemiologia, Assentamentos Rurais, Fatores de Risco, Fatores Socioeconômicos, Estudos Transversais

## Abstract

**OBJECTIVE:**

Investigate patterns and factors associated with tobacco consumption among residents of a rural settlement.

**METHODS:**

A cross-sectional study conducted between September and November 2014, with 172 residents of a rural settlement in the Midwest region of Brazil. We analyzed as dependent variables tobacco consumption at some point in life; current tobacco consumption; tobacco abuse; and the high risk of nicotine dependence, with sociodemographic variables associated with tobacco use, and we applied the Alcohol, Smoking, and Substance Involvement Screening Test (ASSIST) and Self-Reporting Questionnaire (SRQ-20).

**RESULTS:**

The prevalence of tobacco use in life, current use, tobacco abuse, and high risk of nicotine dependence were 62.2%, 20.9%, 59.8%, and 10.3%, respectively. Advanced age, low education level, evangelical religion, marijuana use, hypnotic or sedative consumption, and male gender were factors associated with smoking patterns in the settlers.

**CONCLUSIONS:**

There was a high prevalence of smoking patterns, evidencing the need for public policies on tobacco prevention and control in this population.

## RESUMO

**OBJETIVO:**

Investigar os padrões e fatores associados ao consumo de tabaco em residentes de um assentamento rural.

**MÉTODOS:**

Estudo de corte transversal realizado entre setembro e novembro de 2014, com 172 residentes de assentamento rural, na região Centro-Oeste do Brasil. Foram analisadas como variáveis dependentes o consumo de tabaco alguma vez na vida; o consumo atual de tabaco; abuso de tabaco; e alto risco de dependência nicotínica, com as variáveis sociodemográficas associadas ao consumo de tabaco, com aplicação dos instrumentos *Alcohol, Smoking and Substance Involvement Screening Test* (ASSIST) e *Self-Reporting Questionnaire* (SRQ-20).

**RESULTADOS:**

As prevalências de uso de tabaco na vida, uso atual, abuso de tabaco e alto risco de dependência nicotínica foram de 62,2%, 20,9%, 59,8%, 10,3%, respectivamente. Idade mais elevada, escolaridade baixa, religião evangélica, consumo de maconha, consumo de hipnóticos ou sedativos, sexo masculino foram fatores associados aos padrões de consumo de tabaco nos assentados.

**CONCLUSÕES:**

Verificaram-se elevadas prevalências nos padrões de consumo de tabaco, evidenciando a necessidade de políticas públicas de saúde de prevenção e controle do tabaco nessa população.

## INTRODUCTION

Smoking is a serious public health problem around the world. Tobacco use is responsible for approximately 5.1 million deaths per year, mainly due to chronic noncommunicable diseases (neoplasms, cardiovascular and respiratory diseases)^23^.

In 2013, the World Health Organization (WHO) estimated that 21.0% of adults consume tobacco regularly (1.1 billion people globally). In Brazil, it is estimated that the prevalence of tobacco consumption is 15%^18^. Although there are effective and sound public policies for tobacco abuse and dependence control at the global level, the tobacco industry still promotes strategies to attract vulnerable populations, such as adding flavors and changing the aroma in various tobacco presentations, making the possibility of consumption more pleasant and consequently increasing the rates of tobacco use and nicotine dependence^13^.

Tobacco consumption is increasingly concentrated in certain populations, such as individuals with low income and socioeconomic status^22^. In this context, residents of a rural settlement constitute a population that is highly vulnerable to tobacco consumption. In general, these individuals have unfavorable conditions (low socioeconomic power and low education level) and multiple risk factors for tobacco use, such as family problems, high prevalence of use and abuse of other psychoactive substances (e.g., alcohol and illicit drugs), lack of knowledge about the risks of tobacco use, and difficulty accessing health care^8^ ^,^ ^16^.

Some studies have shown high tobacco consumption prevalence among residents of rural areas^1^ ^,^ ^7^ ^,^ ^9^ ^,^ ^14^. In the United States of America, a study identified that the prevalence of tobacco use in this population ranges from 24.9% to 28.0%^7^. In Bangladesh, a prevalence of 23.6% was found in residents of rural areas^9^. In Malaysia, one study found a prevalence of 56.9% in rural populations, higher than the one estimated in urban residents (45.2%)^14^. In Brazil, a study conducted in rural populations estimated a prevalence of regular tobacco use of 20.3%, a rate higher than the one estimated in urban residents of the country (16.6%)^1^.

In Brazil, there are few studies on tobacco use and dependence in rural settler populations^5^ ^,^ ^19^. Thus, investigating the epidemiology of tobacco consumption in this population group can contribute to actions and guidelines of public policies for tobacco prevention and control in residents of urban settlements, considering the peculiarities inherent to the rural area. Thus, the purpose of this study was to investigate patterns and factors associated with tobacco consumption among residents of a rural settlement.

## METHODS

A cross-sectional, population-based study of residents of a rural settlement located in the southeast of the state of Goiás, in the Brazilian Midwest. The settlement, created in 2005, has a total area of 4,322 hectares, is currently inhabited by 84 families and composed of 250 people (200 adults and 50 children and adolescents). Residents have precarious living conditions, with houses mostly of masonry and they have no treated water, sewage, and regular garbage collection. There is no local health unit. The data collection took place between September and November 2014.

In this study, we included individuals aged 18 years or older who had lived in the settlement for at least six months. Subjects who were not in their residence for up to three occasions of the field researchers’ visits were excluded.

Initially, a meeting was scheduled with the leaders of the settlement to present the study proposal and obtain their consent. Subsequently, a second meeting was held with the residents to explain the research objectives, methods, and benefits to them, and to request voluntary and anonymous participation.

All participants were recruited at home, in the morning or afternoon. After the authorization to enter the residence, the settlers who agreed to participate in the study signed the free and informed consent form and were then interviewed, face-to-face, by previously trained researchers from the project team.

Participants were interviewed using a structured questionnaire on sociodemographic characteristics and factors associated with tobacco consumption. They also answered questions from the instruments Alcohol, Smoking, and Substance Involvement Screening Test (ASSIST)^22^, for the screening of smoking patterns, and the Self-Reporting Questionnaire (SRQ-20), for the detection of common mental disorder (CMD)^10^.

The dependent variables of this study were extracted from ASSIST, an instrument that detects use and problems related to psychoactive substance abuse. It consists of issues related to the frequency, abuse, and risk of dependence on licit and illicit drugs^22^. Scores smaller than zero to three (or zero to 10 in the case of alcohol) identify an exposed person with low risk of presenting problems related to substance use; scores of four to 26 (or 11 to 26 for alcohol) indicate moderate risk, i.e. harmful or problematic use of substances; scores above 27 for any substance suggest that the person is at high risk of addiction^22^.

For this investigation, the following dependent variables were considered: (i) tobacco consumption at some point in life; (ii) current tobacco use, defined by tobacco use at least once in the past 30 days; (iii) tobacco abuse, as defined by a score of four to 26 in ASSIST; and (iv) high risk of nicotine dependence, defined by a score ≥ 27 in the ASSIST evaluation.

The following independent variables were evaluated: age (year), marital status (single or separated; married), gender (female; male), children (no; yes), education (year), religion (none; Catholic; Evangelic), suffered acts of violence (no; yes), regular practice of physical activity (no; yes), access to a basic health unit (no; yes), use of hypnotics or sedatives in the last 30 days (no; yes), marijuana use at some point in life (no; yes), use of cocaine or crack at some point in life (no; yes), and suspected CMD (no; yes).

Age was categorized as: < 30 years, 30 to 44 years, and > 44 years and education was categorized into ≤ 8 years of study and > 8 years of study. We considered a regular practice of physical activity as the individual who reported a frequency of at least 150 minutes of moderate aerobic physical activity (such as walking or gymnastics) or 75 minutes of vigorous aerobic physical activity throughout the week (such as running or soccer), according to the recommendations of the World Health Organization^24^.

Suspected CMD was measured by the SRQ-20, a psychiatric screening instrument validated in Brazil in 1986^17^. It is a questionnaire composed of 20 questions related to nonpsychotic mental disorders in the last 30 days. Each of the items can present as a score of zero or one. The result ranges from zero (no probability of CMD) to 20 (extreme probability of CMD). Scores of seven points or higher suggest the presence of CMD^10^.

The data were analyzed in the Stata Software Package, version 12.0. Prevalence of smoking patterns was calculated with 95% confidence intervals (95%CI). Univariate and multivariate analyses were performed to estimate the factors associated with each of the dependent variables. Initially, a univariate analysis was performed. Subsequently, variables with p < 0.10 were included in the Poisson regression model to obtain the adjusted prevalence ratio (adjPR) and 95%CI. Chi-squared or Fisher’s exact test was used to analyze the differences between the proportions, and variables with p < 0.05 were considered statistically significant.

This study was approved by the Research Ethics Committee of the Universidade Federal de Goiás (Protocol 162/2012, CAAE: 33249014.4.0000.5083) and respected the ethical principles of research involving human beings governed by Resolution 466/2012.

## RESULTS

Of the 84 families in the settlement, 200 residents were considered potentially eligible, according to the inclusion criteria. Of these, seven refused to participate and 21 were not found in their residences during the field investigators’ visits. Thus, 172 settlers participated in the study.

Of the total number of participants, 47.7% were female. The mean age of participants was 44.0 (SD = 14.3) years, and the majority were married (69.2%). Regarding education, approximately half (52.9%) had less than 8 years of schooling.

The prevalence of tobacco use in life, current use, tobacco abuse, and high risk of nicotine dependence were 62.2%, 20.9%, 59.8%, and 10.3%, respectively. Tables 1 and 2 present the univariate and multivariate analyses of the factors associated with these consumption patterns.

**Table 1:** Univariate analysis of factors associated with lifetime and current tobacco consumption in residents of a rural settlement. Brazilian Midwest, 2014.

Variable	Total^a^	Lifetime tobacco consumption		Gross PR		Current tobacco consumption^b^		Gross PR	
				
	(n = 172)	n	%	95%CI	p^b^	n	%	95%CI	p^b^
Age (years)									
< 30	45	13	28.9	1.00		6	13.3	1.00	
30–44	45	30	66.7	2.30 (1.39–3.82)	**< 0.01**	10	22.2	1.66 (0.65–4.20)	0.28
> 44	82	64	78.0	2.70 (1.68–4.33)	**< 0.01**	20	24.4	1.82 (0.79–4.23)	0.15
Gender									
Female	82	47	57.3	1.00		15	18.3	1.00	
Male	90	60	66.7	1.16 (0.91–1.47)	0.21	21	23.3	1.27 (0.70–2.30)	0.42
Children									
No	48	49	39.6	1.00		7	14.6	1.00	
Yes	124	88	71.0	1.79 (1.24–2.59)	**< 0.01**	29	23.4	1.60 (0.75–3.42)	0.22
Education (years)									
≥ 8	81	33	40.7	1.00		9	11.1	1.00	
< 8	91	74	81.3	1.99 (1.50–2.64)	**< 0.01**	27	29.7	2.67 (1.33–5.34)	**< 0.01**
Marital status									
Single or divorced	53	22	41.5	1.00		8	15.1	1.00	
Married	119	85	71.4	1.72 (1.22–2.40)	**< 0.01**	28	23.5	1.55 (0.76–3.19)	0.22
Religion									
No	18	8	44.4	1.00		5	27.8	1.00	
Evangelic	73	42	57.5	1.29 (0.74–2.25)	0.36	6	8.2	0.29 (0.10–0.,86)	**0.03**
Catholic	81	57	70.4	1.58 (0.92–2.70)	0.09	25	30.9	1.11 (0.49–2.51)	0.80
Suffered an act of violence									
No	138	85	61.6	1.00		25	18.1	1.00	
Yes	34	22	64.7	1.05 (0.79–1.39)	0.73	11	32.4	1.78 (0.97–3.26)	**0.06**
Regular physical activity									
Yes	57	26	45.6	1.00		7	12.3	1.00	
No	115	81	70.4	1.54 (1.13–2.10)	**< 0.01**	29	25.2	2.05 (0.95–4.40)	0.07
Access to BHU									
Yes	139	92	66.2	1.00		29	20.9	1.00	
No	15	15	45.5	0.68 (0.46–1.01)	0.06	7	21.2	1.47 (0.89–2.45)	0.12
Suspected CMD									
No	123	71	57.7	1.00		22	17.9	1.00	
Yes	39	29	74.4	1.28 (1.01–1.63)	**0.04**	10	25.6	1.43 (0.74–2.76)	0.28
Use of hypnotics or sedatives^c^									
No	145	88	60.7	1.00		26	17.9	1.00	
Yes	27	19	70.4	1.59 (0.87–1.53)	0.30	10	37.0	2.06 (1.12–3.78)	**0.02**
Marijuana usage^d^									
No	159	94	59.1	1.00		28	17.6	1.00	
Yes	13	13	100	1.70 (1.48–1.91)	**< 0.01**	8	61.5	3.21 (1.79–5.76)	**< 0.01**
Cocaine or crack usage^d^									
No	166	102	61.4	1.00		34	20.3	1.00	
Yes	6	5	83.3	1.35 (0.92–1.98)	0.11	2	33.3	1.62 (0.50–5.26)	**0.41**

BHU: basic health unit; CMD: common mental disorder

Values with statistical significance presented in bold.

^a^ The values differ because some variables are missing.

^b^ Chi-squared or Fisher’s exact test.

^c^ In the last 30 days.

^d^ Lifetime.

**Table 2:** Multivariate analysis of factors associated with tobacco consumption in residents of a rural settlement. Brazilian Midwest, 2014.

Variable	Adjusted PR	95%CI	p
Lifetime tobacco consumption^a^			
Age (30–44 years)	1.74	1.10–2.75	0.02
Age (> 44 years)	1.89	1.21–2.97	< 0.01
Education (< 8 years)	1.46	1.12–1.90	< 0.01
Marijuana consumption^d^	2.18	1.62–2.93	< 0.01
Current tobacco consumption^b^			
Education (< 8 years)	3.43	1.63–4.38	< 0.01
Evangelic	0.24	0.09–0.64	0.01
Consumption of hypnotics or sedatives^c^	2.67	1.63–4.38	< 0.01
Marijuana consumption^d^	4.06	1.91–8.62	< 0.01

^a^ Adjusted for age, children, marital status, education level, religion, regular physical activity, access to a basic health unit, suspected common mental disorder, and marijuana use.

^b^ Adjusted for education level, religion, regular physical activity, hypnotic or sedative use, and marijuana use.

^c^ In the last 30 days.

^d^ Lifetime.

We observed, in a multivariate model, that the factors independently associated to consumption in life were: age from 30 to 44 years (adjPR = 1.74, 95%CI 1.10–2.75); age over 44 years (adjPR = 1.89, 95%CI 1.21–2.97); education of less than eight years (adjPR = 1.46, 95%CI 1.12–1.90); and marijuana use (adjPR = 2.18, 95%CI 1.62–2.93). Regarding the current use of tobacco, the following remained as associated factors in multivariate analysis: education of less than eight years (adjPR = 3.43, 95%CI 1.63–4.38); evangelical religion (adjPR = 0.243, 95%CI 0.09–0.64); consumption of hypnotics or sedatives (adjPR = 2.67, 95%CI 1.63-4.38); and marijuana consumption (adjPR = 4.06, 95%CI 1.91–8.62) (Table 2).

Of the total number of participants, 59.8% (95%CI 50.3–68.6) presented harmful tobacco consumption and 10.3% (95%CI 5.8–17.4) had a high risk of nicotine dependence, measured by ASSIST. It was verified, in a multivariate analysis, that only male gender (adjPR = 1.68, 95%CI 1.14–2.46) remained an independent factor for harmful tobacco consumption. Also, only hypnotic or sedative consumption (adjPR = 7.12, 95%CI 1.79–28.32) was associated with a high risk of nicotinic dependence after the multivariate analysis.

## DISCUSSION

Based on research in scientific databases, the present research in a rural settlement population is unprecedented regarding the tests of dependent variables “tobacco consumption in the lifetime or current” and associated factors. Associated with the application of a screening instrument for use of psychoactive substances, the Alcohol, Smoking, and Substance Involvement Screening Test is indicated by the World Health Organization, especially in the country’s primary care^22^.

This study investigated patterns and factors associated with tobacco consumption among residents of rural settlements in Goiás. Studies have shown higher prevalence of tobacco use in rural areas compared to urban areas^6^ ^,^ ^14^, suggesting the need for health interventions and the constitution of public policies to prevent and control the use of the substance in individuals of those regions.

Tobacco use represents a real dilemma for the health sphere since it causes several harms to the physical and mental health of the user and their family. However, discontinuation of use may lead to a decrease in these harms. The cessation and reduction of damages constitute a complex process, which requires investigations on the seriousness of the damages, and their relation to the time of use, type of consumption, or even other factors related to intense consumption^6^. Therefore, factors associated with tobacco abuse and dependence should be considered in order to propose changes to tobacco cessation in key populations, such as residents of rural settlements.

The prevalence of current tobacco use in the settlers investigated (20.9%) was higher (17%) than the one found in the same population group in the South of the country^19^, and similar to prevalence estimated in the rural population (20.3%, 95%CI 19.1–21.7) and slightly higher than that of urban areas in Brazil (16.6%; 95%CI 16.1–17.1). Some living conditions of this population, such as poor housing conditions and basic sanitation for the families, may increase the risk of damages regarding current tobacco use^19^. On the other hand, we must consider the potential of this population, such as effective participation in social movements and the politicization of young people^5^, which allows for health promotion and social empowerment interventions.

The present study also exposed high prevalence of harmful use (59.8%) and nicotine dependence (10.3%) in the researched settlements. These consumption patterns are responsible for increasing the global burden of pathologies, increasing the risk of chronic noncommunicable diseases, dyslipidemias, diabetes mellitus, osteoporosis, neoplasms, systemic arterial hypertension, and psychiatric comorbidities^4^. Tracking of harmful use and nicotine dependence should be part of health care for residents of rural areas, focusing on the approach and control of risk factors.

In this study, important sociodemographic characteristics were associated with smoking patterns, such as age, education, religion, and gender. In particular, there was an increase in the prevalence of tobacco use in life with advancing age, suggesting a higher risk of consumption in the older age groups. Indeed, in rural areas, experimentation rates and regular tobacco use are higher in adults and the elderly compared to younger age groups^3^.

In developing countries, rates of tobacco use are higher in individuals with low socioeconomic status (income and low educational level), such as residents of rural areas and urban communities (formal and informal urban settlements)^6^ ^,^ ^11^. This higher prevalence in individuals with low socioeconomic status can be explained by, among other factors, the greater probability of not adhering to treatment for dependence and by the low perception of the risks of tobacco use, as well as by the lower support of social and health programs^11^. As seen in this study, the prevalence of lifetime and current tobacco consumption was higher in settlers with less than eight years of education.

In this investigation, the evangelical religion was a protective factor of current tobacco use (adjPR = 0.24). Studies show religious belief as a robust protective factor for the use of psychoactive substances, such as tobacco^15^. Religiousness has positive effects on mental health since it is associated with the promotion of healthy behaviors for health, including cessation of smoking^12^. Some mechanisms are responsible for this connection, such as the social support of certain religions and the promotion of religious moral values, aimed at the psychosocial well-being^15^.

This research found that male gender was the only predictor of harmful tobacco consumption (adjPR = 1.68). Similarly, in developing and developed countries, there is a greater predominance of tobacco use in men than in women. This association can be explained since, in some cultures, tobacco use is seen as acceptable and as a symbol of status and social power for men^1^ ^,^ ^6^.

Co-use of marijuana and tobacco is common in several populations^20^. In this study, we observed associations between marijuana use and tobacco use in life (adjPR = 2.18) and current (adjPR = 4.06), indicating multi use of substances in the settlers. Co-use of marijuana and tobacco enhances physical and mental health damage, including disorders associated with the use of psychoactive substances, worse rates of smoking cessation, and negative psychomotor and cognitive effects^2^ ^,^ ^20^.

Nicotinic dependence is more prevalent in certain groups, such as individuals with disorders related to substance use and mental disorders. Considering a greater genetic susceptibility, the high prevalence can be explained by nicotine’s ability to promote the reduction of some psychiatric symptoms^21^. In the present study, no statistical association was found between CMD and patterns of smoking. However, we found associations between hypnotic or sedative consumption and current use of tobacco (adjPR = 2.67) and high risk of nicotine dependence (adjPR = 7.12), suggesting a higher prevalence of mental disorders and psychiatric symptoms in individuals who smoke.

This study has some limitations. The cross-sectional nature does not allow for the identification of causal relationships regarding the results found. Also, by being restricted to only one local community, it does not allow the findings to be generalized to all rural populations in Brazil. In addition, the data were self-reported, liable for memory bias and for answering certain questions considered morally correct, and may be under- or overestimated. Despite this, the study exposed several factors that increase the vulnerability of rural populations to tobacco use and dependence.

The problems related to uncontrolled consumption of tobacco in rural areas are favored by the difficulty of accessing areas with working health teams, infrastructure, and even difficulties of adherence to public health programs by this population. Smoking cessation is hampered by several factors, such as cultural and habits rooted in these communities^8^, a gap identified in this study.

This study presented relevant characteristics for ascertaining the consumption-individual-locality relationship, necessary to understand the problem of tobacco use in the rural community. It is also shown to agree with the scientific literature regarding the high prevalence found, as well as the association of this habit with sociodemographic variables (age, education, gender, and religion) and consumption of other substances (hypnotics or sedatives and marijuana).

Thus, these findings favor the development of strategies for verification and diagnosis in the health of rural residents, considering that smoking cessation is guided by a range of political, economic, and biopsychosocial factors. The results also showed the relevance of attention to the health needs of this group, with the objective of offering comprehensive care, ensuring prevention of diseases and promoting health and conditions that impact the quality of life of rural community dwellers. In addition, the results suggest the need to propose guidelines for the formulation of public health policies aimed at this population group, considering their nuances and obstacles, as well as boosting new research focused on the rural population.

## References

[1] Almeida L, Szklo A, Sampaio M, Souza M, Martins LF, Szklo M (2012). Global Adult Tobacco Survey Data as a tool to monitor the WHO Framework Convention on Tobacco Control (WHO FCTC) implementation: the Brazilian case. Int J Environ Res Public Health.

[2] Agrawal A, Budney AJ, Lynskey MT (2012). The co-occurring use and misuse of cannabis and tobacco: a review. Addiction.

[3] Azevedo e Silva G, Valente JG, Malta DC (2011). Trends in smoking among the adult population in Brazilian capitals: a data analysis of telephone surveys from 2006 to 2009. Rev Bras Epidemiol.

[4] Benowitz NL (2010). Nicotine addiction. N Engl J Med.

[5] Castro EG, Martins M, Almeida SLF, Rodrigues MEB, Carvalho JG (2009). Os jovens estão indo embora? Juventude rural e a construção de um ator político.

[6] Chockalingam K, Vedhachalam C, Rangasamy S, Sekar G, Adinarayanan S, Swaminathan S (2013). Prevalence of tobacco use in urban, semi urban and rural areas in and around Chennai City, India. PLoS One.

[7] Doescher MP, Jackson JE, Jerant A, Hart LG (2006). Prevalence and trends in smoking: a national rural study. J Rural Health.

[8] Ferrante VLSB, Barone LA, Duval HC (2012). O final de um ciclo? Reflexões sobre assentamentos rurais no Estado de São Paulo. REDD Rev Espaço Dialogo Desconex.

[9] Gfroerer JC, Larson SL, Colliver JD (2007). Drug use patterns and trends in rural communities. J Rural Healh.

[10] Gonçalves DM, Stein AT, Kapczinski F (2008). Avaliação de desempenho do Self-Reporting Questionnaire como instrumento de rastreamento psiquiátrico: um estudo comparativo com o Structured Clinical Interview for DSM-IV-TR. Cad Saude Publica.

[11] Hiscock R, Bauld L, Amos A, Fidler JA, Munafò M (2012). Socioeconomic status and smoking: a review. Ann N Y Acad Sci.

[12] Levin J (2010). Religion and mental health: theory and research. Int J Appl Psychoanal Stud.

[13] Levy D, Jiang M, Szklo A, Almeida LM, Autran M, Bloch M (2013). Smoking and adverse maternal and child health outcomes in Brazil. Nicotine Tob Res.

[14] Lim HK, Ghazali SM, Kee CC, Lim KK, Chan YY, Teh HC (2013). Epidemiology of smoking among Malaysian adult males: prevalence and associated factors. BMC Public Health.

[15] Lucchetti G, Lucchetti ALG (2014). Spirituality, religiosity and substance use: evidence and proposed mechanisms. J Subst Abuse Alcohol.

[16] Mao A, Yang T, Bottorff JL, Sarbit G (2014). Personal and social determinants sustaining smoking practices in rural China: a qualitative study. Int J Equity Health.

[17] Mari JJ, Williams P (1986). A validity study of a psychiatric screening questionnaire (SRQ-20) in primary care in the city of São Paulo. Br J Psychiatry.

[18] Ministério da Saúde (BR) (2016). Plano Nacional de Saúde: PNS 2016-2019.

[19] Oliveira JC, Fadel CB, Lemos JRD, Kuhn WM (2012). Construção de diagnósticos de saúde na agricultura familiar: uma iniciativa à luz do Programa Universidade sem Fronteiras. Rev Cienc Ext.

[20] Peters EN, Budney AJ, Carroll KM (2012). Clinical correlates of co-occurring cannabis and tobacco use: a systematic review. Addiction.

[21] Williams JM, Gandhi KK, Lu SE, Kumar S, Shen J, Foulds J (2010). Higher nicotine levels in schizophrenia compared with controls after smoking a single cigarette. Nicotine Tob Res.

[22] World Health Organization (2003). The alcohol, smoking and substance involvement screening test (ASSIST): guidelines for use in primary care. Draft version 1.1 for field testing.

[23] World Health Organization (2009). Global health risks: mortality and burden of disease attributable to selected major risks.

[24] World Health Organization (2010). Global recommendations on physical activity for health.

[25] World Health Organization (2015). WHO report on the global tobacco epidemic, 2015: raising taxes on tobacco.

